# Case Report: Mutation in *TNNI3*(c. 544G>A): a novel likely pathogenic mechanism of neonatal dilated cardiomyopathy

**DOI:** 10.3389/fped.2023.1291609

**Published:** 2023-11-27

**Authors:** Xianhong Li, Liying Dai, Jian Zhang

**Affiliations:** ^1^ Department of Neonatology, Anhui Provincial Children’s Hospital, Hefei, China; ^2^ Neonate Follow-up Center, Anhui Provincial Children’s Hospital, Hefei, China

**Keywords:** dilated cardiomyopathy, neonate, *TNNI3*, novel mutation, pathogenic mechanism

## Abstract

**Background:**

Dilated cardiomyopathy (DCM) is a rare disease that causes heart failure due to malfunction of the heart muscle characterized by left ventricular dilation and poor systolic function. Genetic screening leads to advantages in early diagnosis and prognostic assessment of patients with suspected inherited cardiomyopathies. Here, we report a case of neonatal dilated cardiomyopathy due to a mutation of the *TNNI3* gene, which has not been published in neonatal dilated cardiomyopathy before.

**Case presentation:**

The patient was a 22-day-old newborn boy with poor ability to respond to stimuli, presenting with shortness of breath over 11 days. He presented with irregular fever, tachypnea, difficulty in ventilator withdrawal, and mild edema of both lower limbs, and III/6SM could be heard in the precardiac area. He presented repeated weaning difficulties during hospitalization with intractable low EF heart insufficiency. Doppler echocardiography showed refractory low ejection fraction, cardiac enlargement, cardiac insufficiency, mild pulmonary hypertension, and mitral and tricuspid insufficiency with mild valve regurgitation. Whole-exome sequencing showed a mutation in the *TNNI3* gene, c. 544G>A (p.Glu182Lys). Thus, he was diagnosed with neonatal DCM. There was no mutation in the parents, the child died 2 weeks after discharge.

**Conclusions:**

*TNNI3* mutation is a novel likely pathogenic mechanism of neonatal dilated cardiomyopathy. Therefore, systematic use of diagnostic tools, advanced risk models, and a deeper understanding of the mechanism are required to reduce morbidity and mortality in this disease.

## Introduction

Dilated cardiomyopathy (DCM) is an idiopathic cardiomyopathy characterized by dilatation of the left ventricle and reduced systolic function, and is a major cause of heart failure and even death (1). DCM accounts for nearly 60% of pediatric cardiomyopathies, and infants under 12 months are at the highest risk (2). Neonatal DCM accounts for approximately 1% of childhood cardiac diseases, and which have an incidence of approximately10 in 100,000 live births (3). There has been limited progress in developing therapeutic strategies for DCM, in part due to the poor understanding of the pathogenesis of the disease. In recent years, studies have suggested that DCM is related to mutations in sarcomeric and non-sarcomeric genes (4). Thus, screening susceptible genes for early DCM intervention may shed light on DCM treatment. In this study, a novel mutation of the *TNNI3* gene was found in a case of clinically diagnosed neonatal DCM through genetic testing. Here, we analyzed the diagnosis, treatment process, and gene testing results of this case to explore new ideas for the diagnosis and treatment of neonatal DCM.

## Case presentation

We report the case of a male neonate born to a G3P3 via spontaneous vaginal delivery at 39 weeks of gestation. His birth weight was 4,350 g, and he had no history of asphyxiation. He had stable vital signs. His body weight was 4,730 g, his length was 55 cm, his head circumference was 38 cm, and his anterior fontanelle was unclosed for 0.2 × 0.2 cm when he was transferred to our hospital on day 22.

The patient had no abnormal symptoms or signs from birth to the 10th day. He developed shortness of breath, moaning, cyanosis and lactation rejection with no obvious cause on the 11th day, and was taken to the local maternal and child health hospital. He was diagnosed with sepsis, neonatal shock, respiratory failure, metabolic acidosis, hyperkalemia, and heart disease, and was given ventilator-assisted ventilation, anti-infection, and symptomatic support therapy for 11 days. However, he had irregular fever, tachypnea, difficulty in ventilator withdrawal, mild edema of both lower limbs, and III/6SM could be heard in the precardiac area. Therefore, he was transferred to our hospital (a tertiary children's hospital) on day 22. On admission, he presented with unconsciousness, poor response, and a pale face, and a few moist rales could be heard in both lungs. His heart rate was 185 beats/min, and III/6SM could be heard in the precardiac area. He had low muscle tone in the extremities, and primitive reflex could not be elicited completely.

Laboratory examinations showed that he exhibited abnormal myocardial enzyme activities in creatine kinase (CK 211 U/L, reference value: 0–200 U/L), creatine kinase-MB (CK-MB 84 U/L, reference value: 0–30 U/L), and cardiac troponin I (CTnI 0.775 ng/ml, reference value: 0–0.06 ng/ml). His lactate value was very high (LAC 587.7 mg/L, reference value 120–300 mg/L). Additionally, he had an abnormal infection index in procalcitonin (PCT 7.160 ng/ml, reference value: 0–0.2 ng/ml), and interleukin-6 (IL-6 30.770 pg/ml, reference value: 0–7 pg/ml). His arterial blood gas, routine blood examination, liver function, renal function, serum electrolytes, bacterial endotoxin, *G*-test, blood culture, sputum culture, and stool tests showed normal results. We also conducted the newborn screening for inherited metabolic diseases, and it did not reveal any abnormalities.

**Figure 1:**
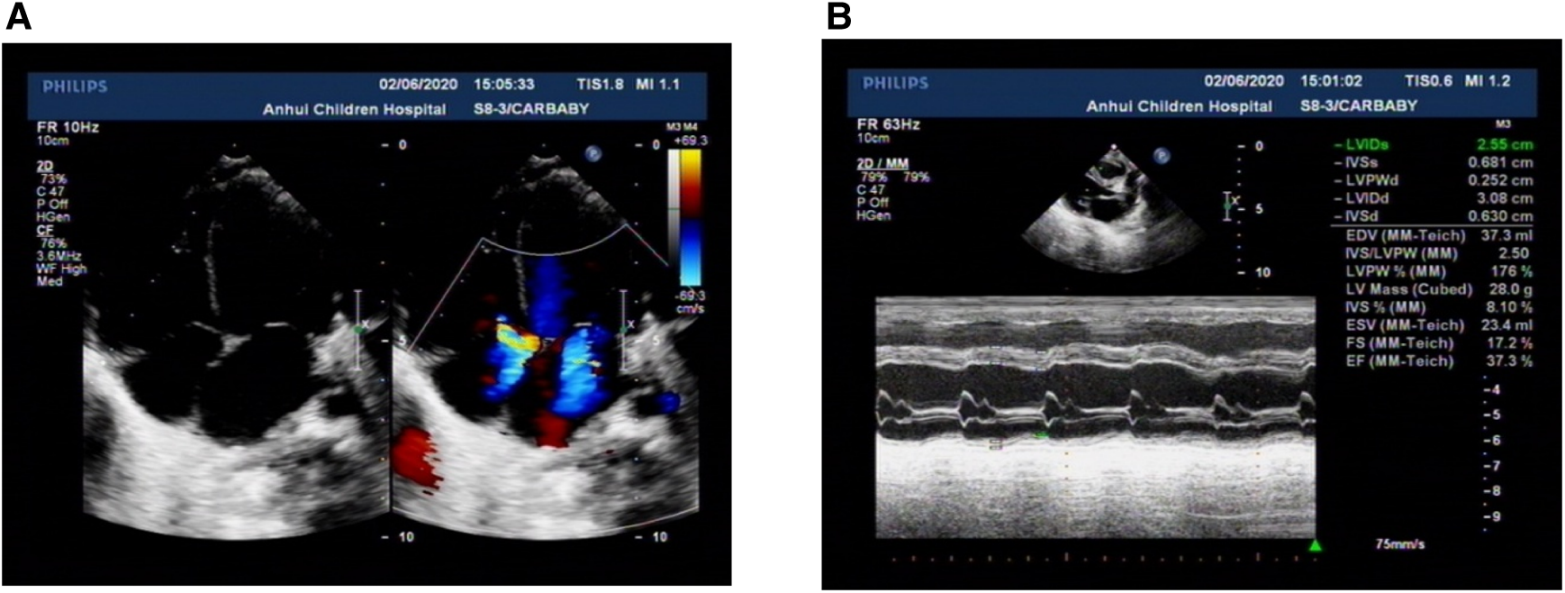
Characteristics of Doppler echocardiography. (**A**) All four ventricles of the heart were enlarged, and the left ventricle was prominent. (**B**) Left ventricular systolic dysfunction was low. All the parents were normal.

The patient could not be weaned off the ventilator for a long time. Therefore, to clarify his pulmonary disease and whether there was a congenital abnormality of lung development, we performed a chest CT scan, and the chest CT showed two pneumonic consolidations with a small amount of pleural effusion, the heart was significantly enlarged, and the main bronchus on both sides was compressed and deformed. Electrocardiography showed sinus tachycardia. CT angiography of the heart showed aortic dilatation and enlarged heart shadow, especially in the left atrium and left ventricle. Doppler echocardiography showed refractory low ejection fraction (EF: 37.3%), cardiac enlargement and cardiac insufficiency, mild pulmonary hypertension, and mitral and tricuspid insufficiency with mild valve regurgitation (Figures 1A,B).

**Figure 2:**
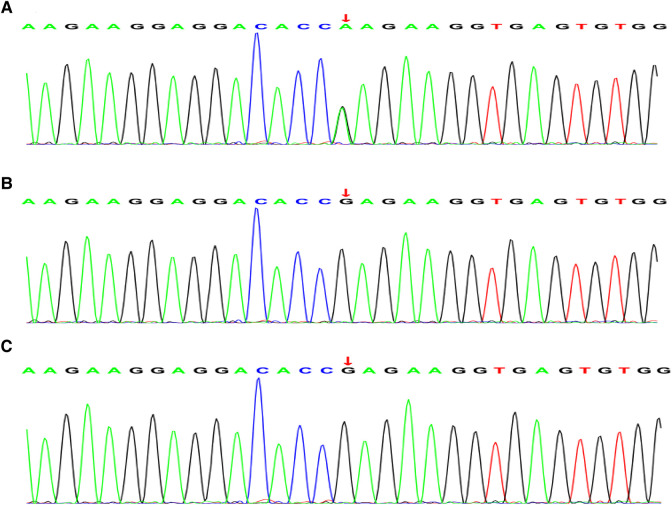
Whole exon sequencing. (**A**) Whole-exome sequencing showing a novel heterozygous missense mutation [c.544G>A(p.Glu182Lys)] in the *TNNI3* gene in the patient. (**B**) (Father) and (**C**) (mother) are all wild-type and normal.

According to trio whole-exome sequencing (WES) of the child and his parents, the result showed a homozygous mutation in 554 based on the 7th exon of the *TNNI3* gene (chromosomal position CHR 19:55665403, reference genome HG19): c.544G>A(p.Glu182Lys) (Figure 2A). This mutation was a missense mutation, that had a very low frequency in the reference population database and was not included in the dbSNP, 1,000 Genomes, and Genome Aggregation Database. Neither of the parents had the same mutation (Figures 2B,C). Combined clinical and genetic evaluations of family members were performed to assess the clinical significance of novel DNA variants, and according to ACMG guidelines (likely pathogenic: PS1 + PM1 + PM2), it was suggested that the harm of *TNNI3* gene variation in this case was related to the phenotype of patient. We also detected other variants in *BTD*, *KCNE2*, and *MTFMT*. However, these genetic mutations did not meet ACMG guidelines and clinical manifestations. Therefore, it supported *TNNI3* mutation was likely pathogenic mechanism of neonatal DCM.

The child was given endotracheal intubation respiratory support for 7 days after admission. Then, after another 4 days of continuous positive airway pressure, oxygen was stopped. PCT, interleukin-6, and cardiac troponin I were normal after2 weeks of treatment with meropenem. Additionally, the patient was given diuretics, fluid restriction therapy, and captopril to reduce myocardial damage and improve cardiac function. After symptomatic treatment, the patient's symptoms showed some signs of improvement. However, the patient's echocardiography still showed cardiac enlargement (02/06/2020 EF: 37.3%, 02/09/2020 EF: 36.0%, 02/14/2020 EF: 36.9%, 02/20/2020 EF: 25.7%, 02/29/2020 EF: 33.4%, 03/07/2020 EF: 32.1%), and the ejection fraction did not increase. Therefore, the possibility of dilated cardiomyopathy was considered. After 34 days in the NICU, his parents discontinued treatment and he was discharged. He died at home 2 weeks after being discharged from the hospital according to a telephone-based follow-up. We speculated that cardiac failure might be the main cause of the child's death.

## Discussion and conclusions

DCM is a cardiac disorder characterized by ventricular chamber enlargement and systolic dysfunction with normal left ventricular wall thickness, which can lead to heart failure and sudden cardiac death (5). The first year after diagnosis is a critical period that is associated with the highest morbidity and mortality. Males are usually less likely to survive to 1 year (6). Clinical manifestations of neonatal DCM commonly include congestive heart failure, dyspnea, congestive pulmonary edema, arrhythmia in some patients, or sudden cardiac death (3). The main clinical manifestations of this child were dyspnea and poor feeding after birth. Echocardiographic and tissue Doppler tests are the basic diagnostic tools for dilated cardiomyopathy in most patients (3). Echocardiography can detect typical left ventricular reverse remodeling (LVRR) changes, and adverse remodeling characteristics in DCM, including functional mitral regurgitation (FMR), myocardial fibrosis, dyssynchronous ventricular contraction, and enlargement of other chambers (7).

A previous study showed that 37% of patients experienced a significant LVRR associated with sudden death/major ventricular arrhythmia in the long-term (8). LVRR is one main prognostic determinant in DCM. Thus, it should be considered a major target in approaching newly diagnosed cases (9), and LVRR is usually related to very poor outcomes. Alexander et al. (10) found that the diagnosis age of DCM was usually less than 4 weeks or greater than 5 years. Familial cardiomyopathy and baseline left ventricular short axis shortening rate *Z* score increased the risk of death or need for heart transplantation. In this study, the patient died within 2 months after diagnosis. During hospitalization, the echocardiography showed persistently low left ventricular ejection fraction and short axis shortening rate.

Molecular genetic studies show that DCM has a familial genetic tendency, and mutations in genes encoding cardiac structural proteins, cytoskeleton, and nuclear envelope proteins are closely related to DCM. Most commonly, familial dilated cardiomyopathy is inherited as an autosomal dominant trait. Autosomal recessive, X-linked, and mitochondrial inheritance patterns are less common (11).

A previous study showed that the mutation genes include the *TTN* gene, *LMNA* gene, Phospholamban gene, and Filamin C gene (1). The *TNNI3* gene provides instructions for making a protein called cardiac troponin I (cTnI), which is only found in the cardiac muscle. cTnI is one of three proteins that make up the troponin protein complex in cardiac muscle cells. Troponin I is the inhibitory subunit of the troponin complex. The troponin complex is associated with a structure called the sarcomere, which is the basic unit of muscle contraction. It complexly contributes to the regulation of contraction of the cardiac muscle (12). Therefore, troponin I is useful in the diagnosis of heart failure. Mutations of the *TNNI3* gene have been confirmed in a variety of cardiac diseases, including hypertrophic cardiomyopathy (HCM), and restrictive cardiomyopathy (RCM) (13, 14). Some evidence has shown that damage of the myocardium could release Ca^2+^. These releases can be induced by mutant cTnI, binding to thin filaments. This has implications with regard to the induction of arrhythmias associated with sudden death in cardiomyopathies (15). However, in contrast to HCM and RCM mutations, DCM mutations in *TNNI3* are less frequent (12). Gomes et al. (16) investigations showed that mutations in cTnI increase Ca^2+^ sensitivity and could lead to a decrease in the ability of cTnI, which inhibits actomyosin ATPase activity, causing impaired relaxation properties and diastolic dysfunction. Robinson et al. (17). Found that changes in Ca^2+^ handling and signaling are common to all mutations, indicating an analogous pathway of pathogenesis of disease in thin-filament sarcomeric DCM. Therefore, this provides an explanation of mutations in proteins that regulate the contractile machinery causing alterations to contraction, calcium handling, and some new signaling pathways for DCM. In this case, a missense mutation of the *TNNI3* gene (c.544G>A) was found through the analysis of the data of the subjects by whole-exome sequencing detection. The mutation is likely a novel mutation by the first generation and has not been reported in the neonate.

Identifying the etiology of DCM in the pediatric population aids risk stratification and prognostication. Approximately, half of cases of DCM have genetically determined autosomal inheritance patterns (18), and approximately 40 causative genes of DCM have been identified (19). As next-generation sequencing technology advances, new mutations are being discovered. Genetic screening has become an important tool for early diagnosis, risk assessment, prognostic stratification, and possible primary preventive measures in such patients.

In conclusion, neonatal dilated cardiomyopathy is a rare disease, and the prognosis is poor. Neonates with dilated cardiomyopathy require rapid diagnosis and therapy to improve their chances of survival. In addition to echocardiography, whole-exome sequencing will improve the early prognosis of the disease. This study reports a new mutation in the *TNNI3* gene for neonatal dilated cardiomyopathy. Therefore, systematic use of diagnostic tools, advanced risk models, and a deeper understanding of the mechanism are required to reduce the morbidity and mortality in this disease.

## Data Availability

The original contributions presented in the study are included in the article/Supplementary Material, further inquiries can be directed to the corresponding author/s.
